# EEG-Based Analysis of the Emotional Effect of Music Therapy on Palliative Care Cancer Patients

**DOI:** 10.3389/fpsyg.2018.00254

**Published:** 2018-03-02

**Authors:** Rafael Ramirez, Josep Planas, Nuria Escude, Jordi Mercade, Cristina Farriols

**Affiliations:** ^1^Music and Machine Learning Lab, Department of Information and Communication Technologies, Pompeu Fabra University, Barcelona, Spain; ^2^Palliative Care Unit, Oncology Service, Parc de Salut Mar, Instituto Mar de Investigaciones Médicas, Barcelona, Spain; ^3^Catalan Institute of Music Therapy, University of Barcelona, Barcelona, Spain

**Keywords:** palliative care, music therapy, EEG, emotion regulation, cancer

## Abstract

Music is known to have the power to induce strong emotions. The present study assessed, based on Electroencephalography (EEG) data, the emotional response of terminally ill cancer patients to a music therapy intervention in a randomized controlled trial. A sample of 40 participants from the palliative care unit in the Hospital del Mar in Barcelona was randomly assigned to two groups of 20. The first group [experimental group (EG)] participated in a session of music therapy (MT), and the second group [control group (CG)] was provided with company. Based on our previous work on EEG-based emotion detection, instantaneous emotional indicators in the form of a coordinate in the arousal-valence plane were extracted from the participants’ EEG data. The emotional indicators were analyzed in order to quantify (1) the overall emotional effect of MT on the patients compared to controls, and (2) the relative effect of the different MT techniques applied during each session. During each MT session, five conditions were considered: *I* (initial patient’s state before MT starts), *C1* (passive listening), *C2* (active listening), *R* (relaxation), and *F* (final patient’s state). EEG data analysis showed a significant increase in valence (*p* = 0.0004) and arousal (*p* = 0.003) between *I* and *F* in the EG. No significant changes were found in the CG. This results can be interpreted as a positive emotional effect of MT in advanced cancer patients. In addition, according to pre- and post-intervention questionnaire responses, participants in the EG also showed a significant decrease in tiredness, anxiety and breathing difficulties, as well as an increase in levels of well-being. No equivalent changes were observed in the CG.

## Introduction

Music known to have the power to induce strong emotions and effectively impact the mood of individuals (Sloboda, 1992; Juslin and Västfjäll, 2008; Koelsch, 2010a). Research involving functional neuroimaging has shown that emotions evoked by music can modulate activity in virtually all limbic and paralimbic brain structures (Koelsch, 2010b, 2014). Thus, music is sometimes used as an adjunct therapy in a variety of clinical conditions (Degli Stefani and Biasutti, 2016; Biasutti and Mangiacotti, 2018). Music therapy (MT) is based on the therapeutic aspects of music. According to the American Music Therapy Association “Music Therapy is an established health profession in which music is used within a therapeutic relationship to address physical, emotional, cognitive, and social needs of individuals” (American Music Therapy Association, 2017). MT techniques may be classified as either *active* (where patients participate actively in the process of music creation) or *receptive* (where patients simply listen to live or prerecorded music). Techniques normally include relaxation/imaginative interventions (receptive), therapeutic use of songs (active or receptive), and various types of improvisation (active). Verbal interaction with the patients can complement MT interventions in some cases but is not strictly necessary (Warth et al., 2014). In general, interventions are personalized according to the needs of the patient (e.g., according to physical state and psychosocial needs).

Helping patients in palliative care and their families to cope effectively with the pain, worries, and emotional impact inherent in the diagnosis of cancer is a recurrent challenge for doctors and nurses in palliative units. In this context, MT may be considered as a candidate for helping to cope and provide emotional and physical comfort to patients and their families. Active MT (e.g., interactive live music performances) delivered by trained music therapists using singing voice and music instruments can engage patients in ways that receptive MT (e.g., prerecorded music) cannot (Standley, 1986; Standley and Hanser, 1995). Studies have found that live music is more effective than prerecorded music with adult cancer patients, i.e., patients over 17 years old (MacGill, 1983). Live MT allows for personalized interactions which may be particularly important for patients who relate best to music which is relevant to their special current situation (Stecher et al., 1972). In clinical palliative care, where the patient’s medical condition is not likely to be improved, the objective of MT is often to improve the patient’s quality of life, e.g., the improvement of pain, stress, and help to regulate negative emotions, e.g., depression, anxiety, anger (Planas et al., 2015), as well as to enhance communication (Warth et al., 2014). MT has been associated with a reduction of anxiety (Nguyen, 2003; Horne-Thompson and Grocke, 2008) and pain (Krout, 2001; Lee, 2005; Curtis, 2011; Gutgsell et al., 2013), in addition to enhancing communication (Brown, 2006) and spiritual well-being (Wlodarczyk, 2007). Hilliard (2003) reported a significant improvement of quality of life in terminally ill patients using MT compared to standard medical care only. Nakayama et al. (2009) reported a decrease in salivary cortisol levels after nine participants received a receptive MT session. Furthermore, MT has been found not only useful for end-of-life patients, but also for family and caregivers (O’Callaghan, 2009). However, current reviews consistently state that there is a lack of rigorous studies providing quantitative grounds for recommending or not the use of MT in the context of palliative care (Korczak et al., 2013; Bradt and Dileo, 2014). The 2010 Cochrane review on MT clinical interventions in palliative care reported that only five trials had implemented (quasi-) randomized controlled designs (Bradt and Dileo, 2014).

Recently, the neural correlates of music-evoked emotion have been investigated by the neuroscientific community using both functional neuroimaging and Electroencephalography (EEG) techniques. In particular EEG brain activity information has been used to detect emotional states in humans (Choppin, 2000; Takahashi, 2004; Bos, 2007; Lin et al., 2010; Ramirez and Vamvakousis, 2012). Patterns of EEG activity have been found to distinguish emotions induced by stimuli with different valence and arousal levels. Asymmetry patterns in frontal EEG activity have been found to distinguish between positive and negative valence, and patterns of overall frontal EEG activity have been found to identify high and low arousal levels (Schmidt and Trainor, 2001; Ramirez and Vamvakousis, 2012). Ramirez et al. (2015) describe an approach to computing in real-time continuous arousal and valence values from EEG activity: based on the EEG signal of a person, the arousal level was determined by computing the ratio of the beta (12–28 Hz) and alpha (8–12 Hz) brainwaves in the prefrontal cortex, while valence values were computed by comparing the alpha power activation levels of the left and right cortical hemispheres.

The aim of the present study is to contribute to the understanding of the emotional effect (estimated by EEG information) of MT in the context of palliative care. More precisely, the study aims to evaluate the effectiveness of a particular MT intervention (a 30-min session including active and receptive MT techniques) for improving the emotional state (e.g., stress, anxiety, anger, and depression) of palliative care patients by analyzing their EEG activity. The patients’ emotional states were estimated before, during, and after MT sessions in order to evaluate the general emotional effect of the MT session, and to assess the emotional effect of particular (active and receptive) MT techniques. With this aim we randomized and assigned participants (*N* = 40) to two groups: the first experimental group (EG) participated in a MT session, while the second (control) group was provided with company. We compared the EEG-based estimated emotional states effect of MT on participants in the EG with the effects of company on participants in the control group (CG). To the best of our knowledge, the present study is the first clinical randomized controlled trial worldwide to examine the emotional effects of MT in palliative care using brain activity information.

## Materials and Methods

### Participants

The research reported in this paper is the result of a collaboration between the Palliative Care Unit (PCU), Oncology Service, Parc de Salut Mar in Barcelona, and the Universitat Pompeu Fabra, Barcelona, Spain. Recruitment, interventions, and data collection are carried out at the PCU. Data processing and analysis was carried out at the Universitat Pompeu Fabra. All patients were assessed for eligibility according to predefined inclusion and exclusion criteria shown in **Table 1**. Forty adults (13 female and 27 male, mean = 69 years old, *SD* = 15) with normal hearing, participated in the study. Twenty of them were randomly selected to participate in a MT intervention consisting of both active and receptive techniques. The other twenty participants were provided with company by the music therapists but no music was involved in their sessions. Patients were randomly assigned to the MT group or to the company group by using the method of randomly permuted blocks. Participants granted their written consent and the study procedures were positively evaluated by the Clinical Research Ethical Committee of the Parc de Salut Mar (CEIC-Parc de Salut Mar), Barcelona, Spain, under reference number: 2015/6078/I. All participants were patients admitted to the PCU.

**Table 1:** Patients’ inclusion and exclusion criteria.

Inclusion criteria	Exclusion criteria
• Admitted to palliative care	• Agony phase (no responsiveness)
• Advanced cancer	• Cognitive impairment
• Understanding of Spanish or Catalan language	• Deafness


	• Restlessness and agitation


### Materials

#### Music Material

Prior to the MT session, participants in the EG were interviewed about their music preferences in order to identify particular pieces to be included in their MT session. Music included both instrumental and vocal pieces in a variety of music genres (both classical and popular music), e.g., Canon de Pachelbel, La Bella Lola, Rien de rien, Hey Jude, Color Esperanza.

#### Data Acquisition and Processing

**FIGURE 1:**
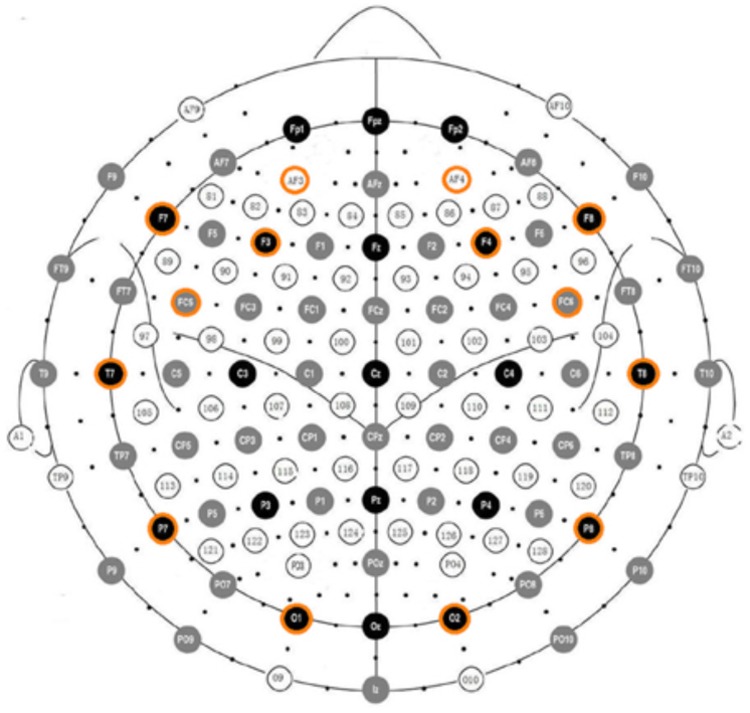
Electrode positions in the Emotiv EPOC according to the international 10–20 system.

The Emotiv EPOC EEG system (Emotiv Systems Inc., 2014) was used for acquiring the patients’ EEG data. It consists of 16 wet saline electrodes, providing 14 EEG channels, and a wireless amplifier. The electrodes’ positions were located at AF3, F7, F3, FC5, T7, P7, O1, O2, P8, T8, FC6, F4, F8, AF4 according to the international 10–20 system (see **Figure 1**). Reference electrodes were placed at P3 and P4 (just above the subject’s ears). Data were digitized using the built-in 16-bit ADC with 128 Hz sampling frequency per channel and sent to the computer via Bluetooth. The obtained EEG data were filtered using Butterworth 8–12 Hz and 12–28 Hz filters. The electrode contact impedance to the scalp was visually monitored using the Emotiv Control Panel software.

The Emotiv EPOC EEG device is a low-cost EEG device, which has been mainly marketed as a gaming device. It captures a lower quality signal compared to the quality of the signal captured by more expensive equipments. However, recent reports evaluating the reliability of some low-cost EEG devices, such as the Emotiv Epoc EEG device, for research purposes suggests that they can be reliable for measuring EEG signals (Debener et al., 2012; Thie et al., 2012; Badcock et al., 2013). A usability review of the Emotiv EPOC EEG device as well as of other low-cost systems can be found in Badcock et al. (2013). For recording and processing the data, the OpenViBE platform (Renard et al., 2010) was used.

### Methods

**FIGURE 2:**
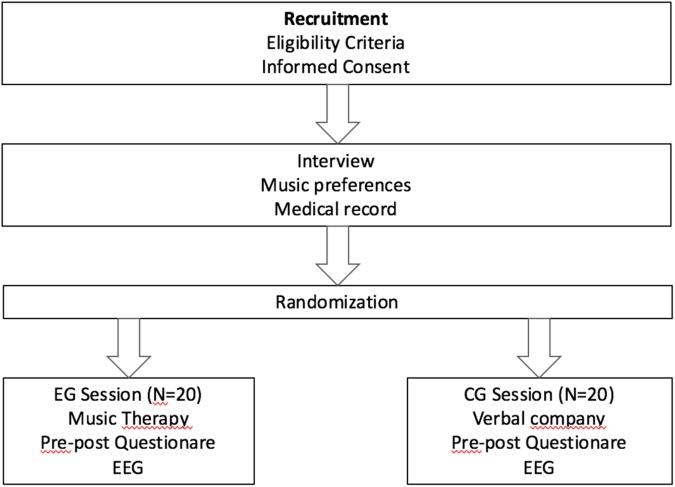
Flow diagram of the study design.

Patients eligible for inclusion in the study were contacted at the Palliative Care Unit (PCU), Oncology Service, Parc de Salut Mar, and informed about the procedures and objectives of the study. Patients received no information about which of the two interventions was the actual experimental condition. If patients agreed to participate, they were asked to sign the informed consent form. Participants were treated individually. Participants in the EG received a MT session of approximately 30 min. The sessions were conducted by three professional music therapists with extensive experience in palliative care. Each MT session consisted of a receptive song, an active song and a relaxation/imaginative receptive intervention. EEG data was recorded before the MT session, during the session, and at the end of the session. Participants in the CG were accompanied by the same music therapists for approximately 30 min in which they conversed feely about music and their music preferences. All participants were receiving similar levels of medication at the moment of the study. **Figure 2** shows a flow diagram of the study design.

In addition to EEG data, participants self-assessed several qualitative variables before and after the sessions by completing the Edmonton Symptom Assessment System (ESAS) pre and post. ESAS (Bruera et al., 1991; Bruera and Macdonald, 1993; Chang et al., 2000) is designed to help in the assessment of nine common symptoms in patients with cancer. The nine symptoms considered in ESAS are: pain, tiredness, nausea, depression, anxiety, drowsiness, appetite, wellbeing, and shortness of breath. The degree of severity of each symptom is rated in 0–10 numerical scale. For each group (i.e., EG and CG) and each rated symptom, data were analyzed by applying a *t*-test of the differences of pre- and post- values.

#### EEG Analysis

**FIGURE 3:**
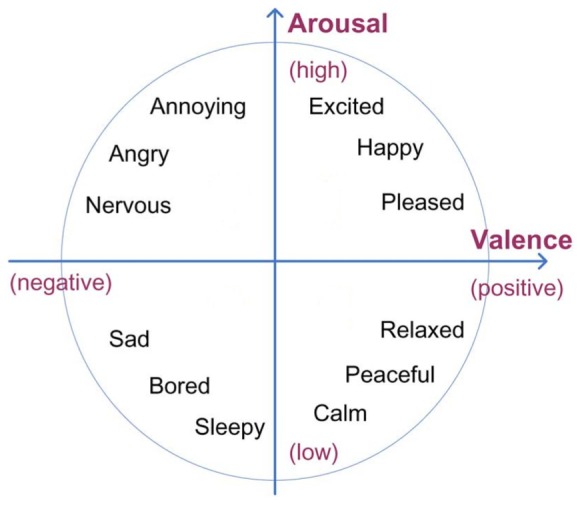
Thayer’s arousal-valence emotional plane.

The patients’ EEG data was transformed into a coordinate in Thayer’s arousal-valence emotion plane (Thayer, 1989), depicted in **Figure 3**. The EEG data processing was inspired by Ramirez and Vamvakousis (2012) where it is shown that the computed arousal and valence values indeed contain meaningful information about the user’s emotional state. Artifact detection/elimination was performed by visual inspection of the signal. EEG data was normalized to avoid inter-participant variability. Using the EEG signal of a participant, his/her arousal level was computed as the ratio of the beta (12–28 Hz) and alpha (8–12 Hz) brainwaves (see Equation 1). EEG data was recorded in 4 locations on the prefrontal cortex: AF3, AF4, F3, and F4 (see **Figure 1**). Beta (β) waves have been associated with alert or excited states of mind, while alpha (α) waves are associated with relaxed or brain inactivation states of mind. Thus, the β/α ratio may be considered as an indicator of the arousal state of a person. More precisely, the instantaneous arousal level of a participant was computed as specified by Equation 1 below:

Arousal=(βF3+βF4+βAF3+βAF4)/(αF3+αF4+αAF3+αAF4)

A number of EEG studies (Henriques and Davidson, 1991; Davidson, 1992, 1995, 1998) have shown that the right hemisphere is more involved in negative emotion while the left frontal area more associated with positive affect and memories. Thus, for computing valence states, in this study we computed the activation levels of the two cortical hemispheres and compared them. Positions F3 and F4 are the most commonly used positions for looking at this valence related activity, as they are located in the prefrontal lobe, which plays a central role in emotion regulation. Valence values were obtained by computing the difference of alpha power α in channels F4 and F3. More precisely, valence level was computed as specified by Equation 2, as following:

Valence=αF4−αF3

## Results

Among the symptoms assessed with ESAS, tiredness (*p* = 0.002), anxiety (*p* = 0.002), breathing difficulty (*p* = 0.042), and wellbeing (*p* = 0.036) showed statistical significant differences (i.e., improvement) between pre and post values in the EG. No statistically significant differences were found in the pre and post values of the qualitative indicators in the CG.

Using the EEG data obtained during both the MT sessions and the company sessions, average valence and arousal values were computed at the beginning and at the end of the sessions (**Table 2**). Average valence values in **Table 2** correspond to the average degree of relative alpha activity in the left frontal lobe, thus larger values are associated with more positive emotional states. Average arousal values on the other hand correspond to either more beta activity or less alpha activity (or both) in the frontal lobe, and thus larger values represent higher arousal states. For the EG, the computed average arousal values (standard deviation) were -0.3 (0.25) and -0.19 (0.18) for the beginning and end of session, respectively, while the computed average valence values (standard deviation) were -0.23 (0.16) and 0.08 (0.17) for the beginning and end of the session, respectively. For the CG, the computed average arousal values were -0.35 (0.25) and -0.24 (0.24) for the beginning and end of the session, respectively, while the computed average valence values were -0.16 (0.38) and -0.11 (0.33) for the beginning and end of session, respectively.

**Table 2:** Average and standard deviation of arousal and valence values at the beginning and at the end of the session.

Group	Indicators	Beginning		End	
			
		Average	*SD*	Average	*SD*
EG	Arousal	-0.30	0.25	-0.19	0.18
	Valence	-0.23	0.16	-0.08	0.17
CG	Arousal	-0.35	0.25	-0.24	0.24
	Valence	-0.16	0.38	-0.11	0.33


In the EG, both the difference between arousal values (*p* = 0.003) and the difference between valence values (*p* = 0.0004) at the beginning and the end of the MT sessions were statistically significant. No significant differences were found in the CG.

**FIGURE 4:**
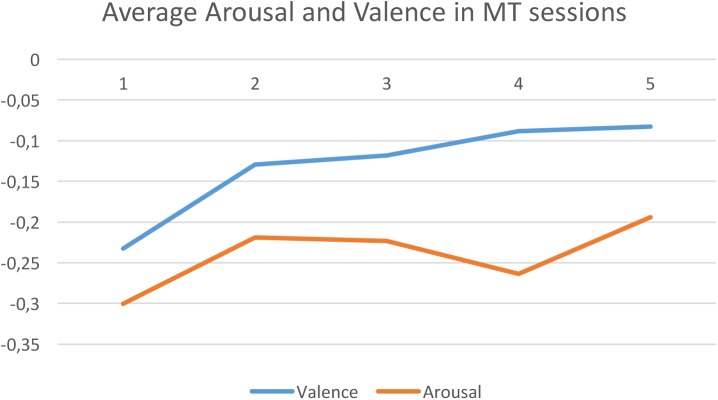
Experimental group averaged arousal (Orange) and valence (blue) levels along time. 1 = beginning, 2 = receptive song, 3 = active song, 4 = receptive imaginative intervention, and 5 = end of session.

**Figure 4** shows the correlation within a session between time and the computed average arousal (orange line) and valence (blue line) values, for the EG. Periods in time correspond to each of the session sections: beginning, receptive song, active song, receptive imaginative intervention, and end of session. For arousal the obtained correlation was *r* = 0.63 (*p* = 0.25) while for valence it was *r* = 0.89 (*p* = 0.04).

**FIGURE 5:**
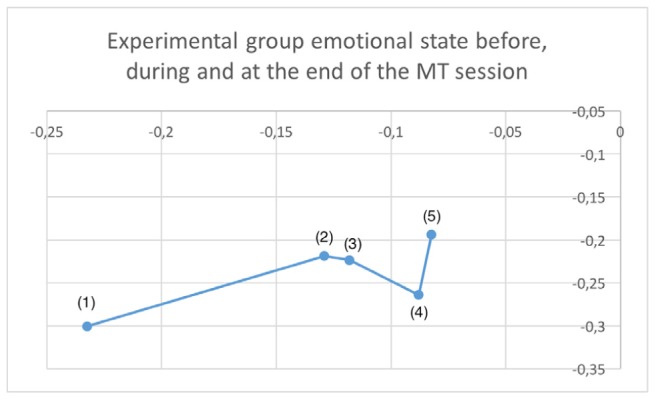
Plot in the arousal/valence plane for the averaged estimated emotional state of participants in the EG during one session: initial state (1), receptive song (2), active song (3), receptive imaginative intervention (4), and final state (5).

**Figure 5** shows the plot in the arousal/valence plane for the averaged estimated emotional state of participants in the EG during the music therapy session: initial state (1), receptive song (2), active song (3), receptive imaginative intervention (4), and final state (5).

In order to investigate if there was a correlation between the participants’ initial and final emotional state, we trained a (regression) support vector machine (SVM) (Cristianini and Shawe-Taylor, 2000) with linear kernel to learn a model to predict the final emotional state of participants given their initial state. Pearson correlation coefficients of the predicted end-of-session values with respect to the real end-of-session values were computed using 10-fold cross validation. For the EG we obtained *r* = 0.53 for arousal and *r* = 0.77 for valence, while for the CG the obtained correlations were *r* = -0.15 for arousal and *r* = 0.13 for valence.

## Discussion

Analysis of the qualitative self-reported data showed that 12 out of the 20 participants in the EG reported feeling less weak after the MT session compared with the beginning of the session (while none of the other participants in the group reported increased weakness), confirming the reported statistically significant difference (*p* = 0.002) between pre and post weakness self-reported values. On the other hand, six out of the 20 participants in the CG reported feeling weaker after the company session (while only two reported feeling less weak). Similarly, 11 out of the 20 participants in the EG reported feeling less anxious, and 12 in a better mood after the MT session compared to their self-reported values at the beginning of the session. This is in line with the statistically significant decrease in anxiety (*p* = 0.002) and increase in mood (*p* = 0.036).

Electroencephalography data obtained showed that overall valence level in the participants in the EG was significantly higher at the end of the MT session compared to the starting level (*p* = 0.0004). This was not the case in the CG where no significant difference in valence levels was found. This result should be interpreted as a decrease of relative alpha activity in the left frontal lobe in the EG participants, which may be interpreted as an improvement of mood or a lessening of depressive mood (Henriques and Davidson, 1991; Gotlib et al., 1999; Ramirez et al., 2015). This reinforces the significant improvement in self-assessment mood reported by the participants in the EG. Similarly, arousal values at the beginning and at the end of the MT session showed a smaller but nevertheless significant difference (*p* = 0.003) in the EG, while no difference in arousal values was found in the CG. The lower *p*-value for arousal may be due to the fact that while most of the patients with terminal cancer are naturally in a low arousal state (e.g., low-mood or depressed), there may be some patients who feel anxious, i.e., are already in a high arousal state. EEG data also showed a significant improvement in valence in participants in the EG reflecting a positive change in their initial emotional state. It is worth noting that while there was a continuous improvement in the participants’ valence throughout the whole MT session, the first MT intervention (i.e., the receptive song) alone produced a significant improvement in valence (*p* = 0.0019) when compared to the EG participants’ initial state.

Regarding the relative effects of the different MT techniques applied during the session (i.e., passive listening, active listening, and relaxation), relaxation produced significantly lower arousal levels than active listening in participants in the EG (*p* = 0.025). This result was expected given that *R* is a relaxation technique used for managing both psychological and physiological agitated states. Surprisingly, no similar significant differences were found between relaxation and passive listening. No relative significant differences in valence were found between passive listening, active listening and relaxation.

In the EG no significant correlation between arousal values and time was found. This may be because of differences between the participants’ states of arousal, as previously mentioned, to the different MT techniques used in the sessions, or the differences between participants’ sensitivity to music. Interestingly, the correlation between computed valence levels and time within the MT session was found significant (*p* = 0.038), which represents a gradual and constant improvement in the EG participants’ valence emotional state. It has to be noted that time and type of MT intervention are confounded, thus this result has to be investigated further in order to establish if it is due to the natural progression of the MT session or to the particular sequence of interventions.

Considering the observed improvements in valence levels in one MT session and the limited duration of each session (i.e., approximately 30 min), it seems possible that further improvement in valence levels may have been obtained if sessions had been longer and/or if treatment had consisted of more sessions. Unfortunately, due to the very short life-span (2 weeks on average) of the participants in the study it was impossible to program more than one MT session per participant. In the past, only a few studies in the literature have investigated the long-term effect of MT. In the current study, no follow-up of the participants in order to examine the long-term effect of MT was possible. We plan to investigate this issue further, perhaps considering a different group of patients.

The question of personalization in MT is an important one but nevertheless it has been little investigated. In this context, we asked ourselves if the emotional state of the participants at the end of the session was related to their emotional state at the beginning of the session. In order to investigate this issue, machine learning techniques were applied to obtain a computational model to predict the participants’ emotional state at the end of the session given their initial emotional state. The accuracy of the obtained models (*r* = 0.53 for arousal and *r* = 0.77 for valence in the EG, and *r* = -0.15 for arousal and *r* = 0.13 for valence in the CG), indicate that there is a moderate/strong relationship between the initial and final arousal/valence states of participants in the EG, while there is no such relationship in the CG. Interestingly, in the context of this study, we showed that it is possible to predict with some degree of accuracy the final emotional state of a person after the MT session based on his/her initial emotional state. This is, using the EEG data of the participants in the study it is possible to extract patterns which allow us to predict the emotional outcome (in particular valence) of new participants after the MT intervention described in this paper. This could open the possibility for personalized MT interventions based on the patient’s state at the beginning of the session. We plan to investigate this further, specifically by adding extra information about the patients (e.g., physiological variables) for training the predictive models.

The results obtained in this study seem to indicate that MT techniques (both active and receptive) can be useful tools for modulating the emotional state of end-of-life patients. Helping such patients to modulate their emotions may improve their quality of life by helping them to cope with the emotional effects inherent in their condition. Although the present study is limited in scope due to the use of only one MT session per participant, it provides an evidence-based rationale for MT in palliative care based on methods involving brain activity (EEG) data. Furthermore, the results obtained open the possibility for personalized MT interventions based on patients’ emotional state before MT is applied.

## Author Contributions

RR supervised data gathering, processed, and analyzed EEG data, and wrote the paper. JP and CF recruited participants and supervised the study at Parc de Salut Mar and contributed to the writing of the paper. NE and JM participated in both the music therapy and company sessions as well as gathered EEG data.

## Conflict of Interest Statement

The authors declare that the research was conducted in the absence of any commercial or financial relationships that could be construed as a potential conflict of interest.
